# Key characteristics and critical junctures for successful Interprofessional networks in healthcare – a case study

**DOI:** 10.1186/s12913-020-05565-z

**Published:** 2020-07-29

**Authors:** Shannon Sibbald, Karen Schouten, Kimia Sedig, Rachelle Maskell, Christopher Licskai

**Affiliations:** 1 Faculty of Health Sciences, Western University, 1151 Richmond Street, London, Ontario N6A 3K7 Canada; 2 Schulich School of Medicine and Dentistry, Western University, 1151 Richmond Street, London, Ontario N6A 3K7 Canada; 3 Western Center for Public Health and Family Medicine, 1465 Richmond Street, London, Ontario N6G 2M1 Canada

**Keywords:** Interprofessional team-based care, Implementation, Networks, Chronic disease, Case study

## Abstract

**Background:**

The use of networks in healthcare has been steadily increasing over the past decade. Healthcare networks reduce fragmented care, support coordination amongst providers and patients, improve health system efficiencies, support better patient care and improve overall satisfaction of both patients and healthcare professionals. There has been little research to date on the implementation, development and use of small localized networks. This paper describes lessons learned from a successful small localized primary care network in Southwestern Ontario that developed and implemented a regional respiratory care program (**The ARGI Respiratory Health Program -** ARGI is a not-for-profit corporation leading the implementation and evaluation of a respiratory health program. Respiratory therapists (who have a certified respiratory educators designation), care for patients from all seven of the network’s FHTs. Patients rostered within the network of FHTs that have been diagnosed with a chronic respiratory disease are referred by their family physicians to the program. The RTs are integrated into the FHTs, and work in a triad along with patients and providers to educate and empower patients in self-management techniques, create exacerbation action plans, and act as a liaison between the patient’s care providers. ARGI uses an eTool designed specifically for use by the network to assist care delivery, choosing education topics, and outcome tracking. RTs are hired by ARGI and are contracted to the participating FHTs in the network.).

**Methods:**

This study used an exploratory case study approach. Data from four participant groups was collected using focus groups, observations, interviews and document analysis to develop a rich understanding of the multiple perspectives associated with the network.

**Results:**

This network’s success can be described by four characteristics (growth mindset and quality improvement focus; clear team roles that are strengths-based; shared leadership, shared success; and transparent communication); and five critical junctures (acknowledge a shared need; create a common vision that is flexible and adaptable depending on the context; facilitate empowerment; receive external validation; and demonstrate the impacts and success of their work).

**Conclusions:**

Networks are used in healthcare to act as integrative, interdisciplinary tools to connect individuals with the aim of improving processes and outcomes. We have identified four general lessons to be learned from a successful small and localized network: importance of clear, flexible, and strengths-based roles; need for shared goals and vision; value of team support and empowerment; and commitment to feedback and evaluations. Insight from this study can be used to support the development and successful implementation of other similar locally developed networks.

## Introduction

The implementation of networks as a strategy to improve healthcare delivery and patient outcomes is burgeoning across Europe and North America [1]. Networks provide connection and promote collaboration between people and institutions [2]; they improve performance and communication [3], support professional development [4], and strengthen relationships [5].

Networks have been researched and significantly developed throughout business-oriented literature [6, 7]. Applying lessons learned from the context of business to healthcare, networks have acted as an integrative and interdisciplinary tool to improve patient outcomes and process efficacies through connecting key players in healthcare teams [8]. Networks have demonstrated improvement in care for chronic disease, mental health, and women’s health [9, 10], across clinical, academic, and public health settings [11, 12]. Also, healthcare networks have been shown to improve patient satisfaction levels [13].

In Canada, several large health-related networks have been created. The Pan-Canadian Public Health Network (PHN) was created to strengthen collaboration and preparedness in public health at an intergovernmental level [14]. Various Primary Care Networks in provinces such as Ontario, Alberta, and Quebec have been created to improve interdisciplinary teamwork [15]. Networks have also been established in research settings to improve relevance of research, such as practice-based research networks (PBRNs). PBRNs are currently used in primary care settings as clinical laboratories to support more efficient and effective primary care research and dissemination [16]. These PBRNs draw insight from healthcare providers to improve patient care, conduct research that is relevant to users, and increase the likelihood of implementing study results into providers’ everyday practice [17].

Despite the ever-growing interest around the use of networks to improve healthcare delivery, there has been little research to date on the development and implementation of small and localized networks. This paper describes lessons learned from the successful implementation of a small localized primary care network to support the development and successful implementation of similar networks in other geographical locations.

## Background

The network studied is a small local network called the Primary Care Innovation Collective (PCIC). The PCIC network currently involves seven Family Health Teams (FHTs) in Southwestern Ontario. FHTs are one of the primary care delivery models found across Ontario at the time of this study (the health system in Ontario is currently undergoing reform; however, primary care continues to be delivered under similar contextual and regional structures during the time of this study).

The PCIC was created in 2009 when several FHTs collectively decided to offer their patients a standardized asthma program, developed by a non-profit organization called Asthma Research Group Incorporated (ARGI). ARGI consisted of a respirologist, a primary care physician, and a respiratory therapist (RT) with a certified respiratory educator (CRE) designation; it developed an asthma program from best practices asthma primary care^1^. When patients were referred to ARGI’s asthma program, the RTs employed by ARGI would contact the patient’s primary care physician to provide the asthma program to augment the patient’s primary care. This specialized asthma program significantly improved patient outcomes, and seven local FHTs collectively contracted ARGI to offer this service as an inhouse program to increase access and to benefit more patients.

The PCIC network consists of a combination of the leadership of the seven FHTs and the founders of ARGI. It holds an advisory role to support the implementation of evidence-based primary care programming within the FHTs and to ensure program fidelity through standardization and a robust continuous quality assurance protocol. As the success of the asthma program offered by ARGI grew, the PCIC began to develop an evidence-based chronic obstructive pulmonary disease (COPD) program to complement the asthma program. ARGI’s Lung health program now offers specialized care for asthma and COPD to the 121,800 rostered patients at the seven FHTs involved in the PCIC network.

The Lung Health Program offers a chronic disease management approach that provides best practice, evidence-based care with a focus on collaborative self-management education for patients to improve self-efficacy – something that is often difficult to achieve with individual practitioners in clinical encounters [18]. The Program uses a multidisciplinary and multifaceted approach to manage chronic diseases through a collection of team-based care interventions; often labeled as an integrated disease management (IDM) model [19]. The Program emphasizes appropriate diagnosis, comprehensive assessment, evidence-based treatment plans, skills training, and disease-specific education and self-management plans for patients. As a team-based care model, the Program supports physicians by reducing the knowledge-to-care implementation gap and improving health outcomes and quality of life for asthma and COPD patients [20].

## Methods

An exploratory case study approach was used to explore network complexity [21]. Site selection was made using Yins ‘extreme case’ rationale [21]: the team chosen for the study demonstrated successful patient outcomes [18], used an innovative chronic disease management model, and expressed a desire to learn more about their team function [22]. While guidelines for chronic respiratory care are available to primary care teams across the province, this ‘extreme case’ has demonstrated consistently successful patient outcomes [18] using their innovative Lung Health Program. Focus groups, observations of team meetings, document analysis and semi-structured phone interviews were conducted to develop a rich understanding of the multiple perspectives associated with the network [23].

Data was collected from four distinct participant groups: [1] members of the PCIC network, referred to in this study as PCIC [2]; RTs working for ARGI that delivered the Lung Health Program, referred to as ARGI [3]; primary care providers (physicians, nurse practitioners, clinical program leads) that work alongside the RTs delivering the Lung Health Program, referred to as ‘Other Providers’; and [4] patients and their family members enrolled in ARGI’s Lung Health Program, referred to as Patients/Family. Focus groups were conducted with groups [1], [2], and [4] and lasted 60 min. Semi-structured phone interviews were conducted with primary care providers (group 3); they lasted between 10 and 25 min. The research team observed network meetings to better understand network function and team dynamics. Interview and focus group guides were developed for this study using the Consolidated Framework for Implementation Research [24] and in consultation with the research team and participants. Observations, collected as field notes, took place at two PCIC meetings and one ARGI meeting. Document analysis was conducted using over 25 documents (such as memorandums of understanding, team meeting minutes, and mission/vision statements) provided by the network.

The data was analyzed iteratively using a modified thematic approach [25]. First, data sources were analyzed by researchers independently to identify concepts and ideas. In this first stage, the analysis process was open and inclusive to create a comprehensive coding list. Next, data sources were analyzed in aggregate to explore emerging themes. These themes were then defined and grouped (coded) into broad categories. Lastly, researchers discussed themes and patterns to create a common coding framework and consistent definitions [26]. Document analysis was incorporated at this stage both to support and validate our findings as well as to ensure congruence of themes. Member checking was done with the PCIC participants (group 1) and the ARGI participants (group 2) by creating an interim report with draft findings and meeting with teams to discuss results. Feedback was incorporated into our findings. Analysis was organized and supported using qualitative analysis software (NVivo11).

Each participant provided written consent prior to participating. REB approval was obtained through Western University’s Research Ethics Board (#108415).

## Results

In total, 40 participants engaged in the study. Participants included *n* = 12 from group 1 (PCIC); *n* = 8 from group 2 (ARGI); *n* = 7 from group 3 (Other Providers); and *n* = 13 from group 4 (Patients/Family) (see Table 1). Our analysis of the data revealed that several key characteristics and critical junctures have supported the successful development and implementation of this small local primary care network. The key characteristics identified include: growth mindset and quality improvement focus; clear team roles that are strengths-based; shared leadership shared success; and transparent communication(see Table 2).

**Table 1:** Breakdown of Study Participants

Data collection method	Participants	Number of participants	Number of times data was collected
Focus Group	Providers PCIC	12	4
	Providers ARGI	8	
	Patients and family members	13	3
Phone Interview	Providers that worked alongside RTs	7	7
**Totals**		**40**	**14**

**Table 2:** Overview of Results and Lessons

Key Characteristics	Critical Junctures	General Lessons
Growth mindset and Quality Improvement (QI) focus	Acknowledging a shared need	Ensure both clarity and flexibility in strengths-based team roles.
Clear team roles that are strengths-based	Creation of a common vision	Develop a common vision and shared understanding of how to get there.
Shared leadership, shared success	Facilitating empowerment	Facilitate ownership and empowerment of the process and the outcomes
Transparent communication	Receiving external validation	Embed evidence gathering, self-evaluation, and feedback mechanisms.
	Demonstrate improvement of patient outcomes	

### Growth mindset and quality improvement focus

Members of the PCIC network shared common characteristics of a growth mindset (i.e. dedication and hard work will produce desired results) and a desire to ensure that the care delivered was of a high quality and included evidence-based practices. This was evident in three ways. First, there was a shared conviction to ensure all programming was best practice and evidence-based. PCIC members took the initiative to review new and emerging best practices, disseminate, and incorporate into the Lung Health Program where appropriate. The PCIC members regularly led “guideline days” where best practice guidelines were reviewed with the ARGI team and experts were often invited as speakers. Second, sharing lessons was a key feature of all team meetings. Learning from one another was a priority, alongside taking time to work together to solve problems. Program improvements were determined collectively by the network through regular reporting on network-wide program use and continuous quality improvement efforts. Since the network’s inception in 2009, the PCIC network has grown from four original FHTs to the current seven. The PCIC also increased the scope of ARGI’s Lung Health Program from a focus solely on asthma to include COPD, and with plans to add heart failure and atrial fibrillation care after clinical trials have been completed. Third, network members actively sought out to evaluate their program. PCIC developed a point of services eTool unique to ARGI that evaluates program fidelity and quality by tracking patient outcomes and program referrals allowing for the network to make data-driven decisions to improve the program. ARGI and PCIC team members collaboratively review challenging cases and provide feedback. The network’s growth mindset and data-driven decisions helped balance program fidelity with flexibility and overall program commitment.

### Clear team roles that are strengths-based

Members of the PCIC network and the ARGI staff identified role clarity as an important factor for their overall success. Participants believed strong role clarity ensured all network members were valued and aware of their role within the network. RTs hired by ARGI were empowered by PCIC to work to the fullness of their scope of practice.

RTs within ARGI understood their roles and shared a common identity as ‘the RTs of the program’. They were proud of the services they offered to patients within the FHTs. This was evident in examining the structure and relationship of the PCIC network with the ARGI team. ARGI RTs were not employees of the FHTs; they were employees of ARGI. Therefore, while RTs felt connected to the FHTs where they provided care, they were more connected to each other through ARGI. RTs met frequently to share challenges and ideas and continuously shape and refine their roles within the FHTs, allowing them to feel ownership over their role. This was augmented by input and oversight from the PCIC where the RTs ideas and challenges were venerated and discussed. The role of PCIC was not to provide care, but to ensure RTs could effectively do their job. The deliberate structural design of ARGI and PCIC complemented one another and facilitated team-wide understanding of roles and limited any redundancy or duplication. This meant all team members felt they had a specific role, and they felt capable of fulfilling it. One participant explained, “I had the same kind of values and team aspect where it’s not just me or her, it’s (the) team” (Provider FG#6). Other providers also acknowledged the role of the RTs as key providers and part of the patient care team, with specialized skills and training. Physicians described how RTs alleviated some of their workload by assisting in consultations and idea sharing for patients with COPD and asthma. Because of the clear roles and confidence in their full scope of practice, the RTs were trusted to provide care in the patients’ best interest. One physician explained how knowing that the RT can support essential elements of respiratory care, allowed the provider to focus on the patients’ medical needs beyond respiratory care. Participants from all four groups identified the competency of the RT team as a contributing factor to the network’s success.

### Shared leadership, shared success

The network is led by inspiring, dynamic leaders. Many participants credited the leadership with creating a positive, empowering culture. Several participants described this as the result of equal and non-competing power dynamics, enabling the team to function optimally:

“…we don’t have that hierarchical structure, we have more of a flat line I believe, when it comes to the allied health professionals and physicians in our model. There is that flat line respect and a flat line understanding that there’s a fit, you’re doing what is helping me, right. It’s like part of it, you’re part of the team” (Provider FG #2).

In defining their team, PCIC network participants articulated both an informal operational structure and a formal administrative structure. A “Vision of Values” was created to guide practice decisions,^1^ whereas more formal memorandums of understandings between the FHTs in the network provided structure around the roles and responsibilities of team members, including resource-sharing agreements.

The leadership and governance model was explained by one provider who said: “… It’s a very flat, flat organizational chart […] And we don’t have administration bearing down on us […] And I would say none of us in the room are people who would slack at all” (Provider FG #1). Policies developed by the PCIC were adapted and tailored to meet the needs, resources, and abilities of the individual FHTs. FHTs’ executive directors and lead physicians on the PCIC were supported and encouraged to use a ‘bottom-up’ approach to solve problems in a context-dependent manner, which fostered continued buy-in. Patients and family members who described their experience viewed the RTs as members of FHTs and did not see a division in the provided healthcare services. The shared development of the network led to shared success of the entire health team. Their co-creation of vision and goals led to shared commitment and ultimately shared success.

### Transparent communication

Participants across all groups described how open and transparent communication was an essential characteristic of the success of the Lung Health Program. PCIC participants highly valued the openness of meetings as well as the shared problem solving. RTs discussed their comfort with being able to discuss “anything” with the leadership, knowing their input was valued. Similarly, Patients/family members acknowledged the paramount role of communication between RTs and physicians. One Patient participant expressed his gratitude for this communication: “You know, the feedback from one (RT to) another. One of my biggest fears is, am I doing okay? Is everything okay? Is there any issues? The feedback mechanism is so strong that I don’t think something (bad) would be left to progress” (Patient FG #7).

#### Critical junctures

Participants experienced and described five critical junctures in their solidification as a network (see Table 2).

### Acknowledging a shared need

PCIC was developed on the acknowledged need to share resources and garner support for quality improvement in primary care. PCIC participants all agreed that the need to work together was very strong and was the driving force behind their performance and success: “we figured out we could do more than what we were just doing. You know, we were brought around for something and it was just so exciting that it was like everybody came, the conversation never stopped, it just kind of, and it was like wow! We should make this bigger” (Provider FG#1). There was also an internal drive to share best practices coupled with an external pressure to provide better care in different ways. The creation of the PCIC Network played a critical role solidifying a shared vision for the respiratory health and gave the Lung Health Program overall legitimacy.

### Creation of a common vision

The “Vision of Values”^2^ along with Memorandums of Understanding agreed upon by the FHTs in the network provided a common vision for the roles and responsibilities of all network members that also outlined resource-sharing agreements. The PCIC network had a shared ideology regarding their chronic disease prevention and management model:

...not all the FHTs were totally developed or had clinical leads or educators in-house, so what were going to be our common goals, and all of that sameness brought us all together trying to mine things so that we weren’t recreating the wheel, ..., what best practice is out there, what other teams are doing and do we have the resources to leverage where we want to go. (Provider FG#1).

The network’s vision provided a common foundation and a shared approach, allowing each member to work towards a collective goal. PCIC and ARGI participants described how the network leaders communicated the vision clearly and helped set goals collectively while continuously supporting individual network members and RTs: “… it’s a very goal-oriented group that looks for solutions and that I find, quite uplifting. Wednesday I’m feeling kind of low and scattered, but Friday (after our team meeting) a little more feeling good, I’ll feel energized, like we’ve got a direction.” (Provider FG#1). For network members, this translated back to their primary goal of delivering high quality patient care. One PCIC participant highlighted the importance of this by describing the improved continuity of care from the Lung Health program: “You want to be able to walk into a family health team and sit down and have the confidence that the same level of care is been provided” (Provider FG#7).

### Facilitating empowerment

Participants across all data sources discussed feeling empowered in their role in the network and the Lung Health program. The PCIC network members described being empowered through the trust that was developed that allowed them to take risks and try new things. One PCIC participant explained, “once we had some experience and some time working together and a trusting relationship, and that freeness and openness of sharing then it just sort of allowed us to grow, mature, and branch off into other areas.” (Provider FG#1). The RTs were empowered through training, collaboration, and mentorship to work independently to their full scope of practice allowing them to feel confident in their role. Further, the RTs ideas are reported in PCIC network meetings and contribute to network decision making. Other Providers felt empowered in their role collaborating with RTs to provide their patients with high quality respiratory care. Physicians trusted RTs to bring best practices into their clinic and support patient care.

Patient participants in turn felt empowered through receiving timely care that focused on collaborative self-management. Arming patients with knowledge and skills about their disease provided confidence that they can manage their symptoms and if they find themselves in trouble that they can rely on their triad to provide additional supports.

### Receiving external validation

The PCIC network received several accolades from external sources for the network’s functioning and program achievements which provided validation to the network. The Ontario Ministry of Health and Long-Term Care acknowledged the excellent achievements of the network and provided funding to support program expansion.

… the asthma research group had completed a lot of work in asthma and was approached by the Ministry [of Heath] […] you’re doing great work so why don’t you do more great work. And it was bringing those two tables together really to say ARGI’s got some great content expertise and some great methodology in terms of how they do things, and a great way of doing asthma; maybe that could push out into a bigger chronic disease (PCIC participant FG#2).

The success of the Lung Health program has been presented at conferences worldwide and published in peer-reviewed journals [18, 27]. They also received honourable mention for the 2013 Minister’s Medal awards and the award for Best Primary Care abstract at the European Respiratory Society in 2016. As the network received external validation, their work became recognizable and they were approached by primary care providers outside of the network for knowledge sharing opportunities. This in turn strengthened the network and provided a sense of pride for everyone involved.

### Demonstrated improvement of patient outcomes

While this study did not explicitly collect patient outcomes data, participants frequently highlighted the success of the network as observed through its achievement of its primary goal: improving patient outcomes. Improving patient outcomes was seen a driving force behind the continued improvements and expansion of PCIC and ARGI. When asked about measuring and evaluating success, participants agreed: “… for us (it’s) a simple answer: our patient outcomes, (and) that’s a result of this group and our teams. That’s a really easy one.” (Provider FG#1). Patient outcome data was comprehensively tracked on their e-tool and regularly discussed at all team meetings. The network boasted about its role in improving the quality of life of more than 2500 patients living in Ontario, Canada suffering from COPD [18]. Participants were proud to explain how these outcomes supported health system performance improvements across the region, seen through the reduction of acute care services including ED visits and hospitalization [28]. Through this, the network believed it provided better outcomes for COPD patients at a lower cost. Ultimately, network participants were proud to be supporting providers, and patients to improve their care experience: “it is based around the patient centered experience and start there because ultimately that’s what it’s about” (Provider FG#2).

## Discussion

This paper aimed to share the lessons learned from the development and implementation of a small localized primary care network. This network was chosen for the study because of the demonstrated success in improving patient outcomes and their desire to learn more about themselves as a network. The results of the study revealed a complementary and integrated network structure that supported and enabled professionals associated with the network to work to the fullness of their skill set. The network structure provided oversight for the delivery of a successful Lung Health Program, which in turn fostered empowerment and pride for all involved.

The results point to four important and generalizable lessons about the use of small, localized networks to support program success (see Table 2).

First, ensuring both clarity and flexibility in individual and team roles is extremely important. Further, in this study, roles that were strengths-based seemed to support the success of the network by building trust across providers and supporting buy-in for the common vision. When individuals understand their role in a team, they are often more able to contribute their own expertise to the team and have room to grow within their roles [29]. This type of strengths-based approach can facilitate a team in addressing complex problems at the clinical interface that considers local needs [30]. It was clear that in this study, network members were encouraged to collaboratively solve problems using innovative solutions, and to discuss mistakes made and lessons learned. Caldwell and O’Reilly support this idea, stating that a group’s ability to successfully implement change can be augmented by a group’s support in risk taking and tolerance of mistakes [31]. It was clear that network members trusted one another and fostered *psychological safety;* members understood their role and felt safe to take risks (try new things, learn from mistakes). Psychological safety has been shown to drastically improve network performance and outcomes [32, 33]; in this case, it supported the implementation of innovative and emerging practices. Literature shows that filling organizations with people that value innovation is more likely to support change efforts [34]. Developing trust and psychological safety requires time and deliberate consideration of the strengths and goals of each team member to cultivate alignment and ownership. This also allows for better role clarity by creating the opportunity to define and refine individual roles. Diversity of thought has been associated with improved task performance in groups [35]. Everyone in the PCIC network knew the roles of others and shared a common goal of improving patient outcomes. While roles were often defined by position within the network (e.g. executive director or RT), individuals could also expand their role based on interest and expertise. Ensuring members of a team or network have a common understanding of everyone’s tasks and roles can uncover beliefs and knowledge discrepancies which can be addressed to improve the function of a network [36].

Second, critical junctures experienced by the networks (agreeing on problems and co-creating solutions) were more likely to promote successful development and implementation when they grew out of on a common vision. This was augmented by having agreement on the process of getting to a common vision. This has been captured in the literature through decision-making frameworks such as ‘accountability of reasonableness’ which purport that agreement on the process is just as, if not more, important than agreement on the outcome [37].

Third, in our study, it was clear that facilitating ownership and empowerment among members of the network improved overall success of network development, implementation and performance. Within the PCIC network, knowing that all members of the network are supported and that each team member has a say in the network’s direction fostered trust. The practice of open contribution, and using “yes, and ...” to facilitate healthy conflict management while making strategic decisions, has been suggested to support network success [35]. Success of the ARGI program (i.e., improved patient outcomes) was crucial for supporting the strategic goals of the network. PCIC used several strategies to monitor their success, such as tracking the number of referrals and the eTool to measure patient outcomes. They also provided opportunities to collaborate on challenging cases. Regular reporting and discussions at ARGI meetings ensured high program fidelity. The network used a shared leadership approach and ensured all members had a voice. Concerns unique to each member/FHT were shared with the group and used as learning opportunities to facilitate collaborative problem-solving. This idea is supported by Aghina, Handscomb, Ludolph & West’s [35] healthy conflict premise, as well as by Allender et al. [38] when they suggest a network can help ensure projects are informed by high quality, best available knowledge, evidence, expertise, and experience.

Fourth, processes for both gathering and recording evidence, as well as robust mechanisms for feedback and evaluation, should be embedded within the network. Creating a network allowed the FHTs to pool their resources, as well as share data to improve decision-making. FHTs in the network were able to work with the RTs to adapt the Lung Health Program to meet the needs of their patients and the resources of the FHT. Sharing data from the eTool allowed best practices to be monitored and maintained across the network. Regular team meetings amongst PCIC and ARGI enabled members to share their experiences and provide support for each other. This suggests that the network’s success is influenced by effective coordination and communication across both operational and strategic activities. This finding is reflected in literature that supports the use of networks to support increased interdisciplinary collaboration, knowledge sharing, and better implementation of evidence-based practice [9]. New leadership within PCIC and new RTs within ARGI, are taught by experienced network members who take on mentorship roles, which enabled constant feedback and further supported program fidelity. This idea is similar to a community of practice model [39], where networks can be used to support the learning of newcomers to support capacity building and implementation of evidence-based practices.

Through the application of these lessons, the network has experienced success in their development and implementation. They have grown and matured as a network and are now working to scale and spread their model both to other diseases (such as heart failure) and to a broader geographic region. This growth has been coupled with continued improvements in the health outcomes of patients enrolled in the Lung Health Program (as demonstrated through a control trial conducted by the network) [18]. The network facilitated the implementation of an integrated disease management model (the Lung Health Program), which led to improved patient care and the substantial reduction of rates of acute care services such as ED visits and hospitalization [18]. Overall, the network delivered a dramatically improved quality of life (QoL) when compared with the mean QoL outcome among COPD and lung health patients. A QoL responder analysis showed a 90% improvement in QoL, and reduced burden of health services use [18]. This is echoed in the literature where patients supported by teams in a strong network reported better management of their symptoms and greater knowledge about how to manage their condition [1]. Long-term, the network has plans to study the cost-effectiveness of the program; reduction in health services utilization is often a good indicator of reduced cost to the system [40]. This in turn will support future implementation of the Network, the Lung Health Program and ultimately lead to sustainable healthcare networks in other geographical locations across the country.

Both PCIC and ARGI demonstrated a commitment to improving quality of care through research and self-evaluation. The network provided a foundation for conducting research, and ensured decisions are informed by their own evidence. The network also empowered members to consider and implement new ideas while reflecting on their outcome and learning from mistakes. In this way, PCIC and ARGI continuously learn and adapt to meet the needs of their patients.

Due to the nature of the research conducted, there are several limitations including the potential for self-report and/or social desirability bias. It is often difficult to verify the results of qualitative research; we used multiple strategies to augment the rigour of our research and trustworthiness of our findings. Our research team was diverse, we collected data across multiple sources, and multiple researchers supported each phase of data collection and analysis. We conducted frequent member checks, and ‘report-backs’, where we presented our findings to our participants to check the accuracy of our work. Case studies take time to conduct; the research team spent a significant amount of time in the field, to develop a deep understanding of the case and participants. Collecting primary data on patient outcomes may have strengthened our findings; however, we were able to gain an appreciation for patient outcomes both through direct conversation with patients and by discussion outcomes with the providers. Since this study focuses on the work of one site, a possible limitation may include the lack of generalizability with the results or interpretations of our findings. However, the lessons learned from this program are not meant to be generalizable; but rather, to be taken as an exemplar for future initiatives involving interprofessional collaboration in healthcare.

## Conclusion

Networks are used in healthcare to act as integrative, interdisciplinary tools to connect individuals with the aim of improving processes and outcomes. In this context, we have identified four general lessons to be learned from a successful small and localized network: importance of clear, flexible, and strengths-based roles; need for shared goals and vision; value of team support and empowerment; and commitment to feedback and evaluations. When a network is set up well, it supports better patient care and improved satisfaction both of patients and healthcare professionals. From our study, the network found success by experiencing five critical junctures: acknowledge a shared need; create a common vision that is flexible and adaptable depending on the context; facilitate empowerment; receive external validation; and demonstrate the impacts and success of their work. Through the application of key learnings from the Lung Health Network, to other chronic diseases, such as heart failure and atrial fibrillation, similar networks could be developed and sustained to benefit other patient populations.

## Supplementary information

**Additional file 1.** Patient & Provider Focus Group/Interview Guides.

## Data Availability

The datasets generated and/or analysed during the current study are not publicly available due to the individual privacy parameters of the study participants as outlined in the research ethics board protocol but are available from the corresponding author on reasonable request.

## Abbreviations

ARGI: Asthma Research Group Incorporated
COPD: Chronic Obstructive Pulmonary Disease
CRE(s): Certified Respiratory Educator(s)
FHT(s): Family Health Team(s)
IDM: Integrated Disease Management
PCIC: Primary Care Innovation Collective
PHN: Public Health Network
PBRN(s): Practice-based Research Networks
QoL: Quality of Life
RT(s): Respiratory Therapist(s)

## Footnotes

1 **Vision of Values of PCIC - Aspiration:** To lead innovation in chronic disease prevention and management. To build a program exemplar for Chronic Disease prevention and management (CDPM) in primary care. **Shared Values:** Patient-centred holistic care, strive for best patient care / best experience**,** proactive primary care upstream from acute care**,** pragmatic health system transformation, shared innovation / shared resource, research and robust program evaluation
