# Supplementary material for: Key characteristics and critical junctures for successful Interprofessional networks in healthcare – a case study
Source: BMC Health Serv Res. 2020 Jul 29;20:700. doi: 10.1186/s12913-020-05565-z (PMC7391486; doi:10.1186/s12913-020-05565-z)

**Patient & Provider Focus Group/Interview Guides**

**iCOPE Provider Focus Group Guide**

SCRIPT:

As you know you have been identified as being a part of a successful, or high performing team. The literature defines a high performing team as being known as innovators, with a reputation for excellent outcomes around better health, improved patient experience, or more affordable cost. Our evaluation of your team started earlier this year. In March, we came to present on our

research, and to observe your team. We had you complete surveys and a drawing, or map. Today, we want to explore some of our findings with you, and learn more about the functioning of this team. Our end goal is two-fold: first, we want to be able to celebrate the successes of this team in a systematic way; second, we want to try to learn from your group, to be able to share lessons to other groups who are trying to implement a similar process.

The purpose of the focus group is for you to discuss together, and build off one another’s ideas. We are here to moderate the discussion, and keep the conversation moving in the right direction. There are no wrong answers.

When we came in March we had you sign a consent form, so we do not need to obtain your consent again today – unless you were not here (ASK: anyone?). We will be audio-recording today’s session, and we have an observer who will be taking notes. The transcript of today, and the notes will be anonymous and confidential. Like all our data, you will have a chance to review our aggregate findings before any publication. Does anyone not want to be recorded?

We are going to start with ‘GRAND TOUR QUESTIONS’.

1. Tell me about how this group started as a team.

a. What were some of the defining moments?

b. Do you remember any roadblocks?

• How were they handled? (conflict resolution)

c. What about facilitators?

2. What role does context play in your team?

a. How did the role of context shift or change from the beginning (developing) to now?

1. Define a successful team.
2. How would you know if a team is successful?
3. What other factors have facilitated or stifled your team’s success?
4. How might you formally measure/monitor/evaluate this success?
5. How might you take steps to improve likelihood of success?

5. The mapping exercise we did was based on an approach to data collected called 'Rich Picture'. Rich Pictures are used in qualitative research to gather information in complex team environments. In our analysis of the maps we found three central themes:

a. The context in which PCIC is situated in complex, but seems to be driven by QI;

b. There are obvious (and sometimes named) leaders to the team. Also important is

collaboration and idea sharing;
c. Outcomes or impacts for patients and families is important.

We're interested in your thoughts on those three themes. Do they resonate? Is anything missing?

6. Let’s talk specifically about the “ARGI” piece of your team.

1. How is it integrated or adapted?
2. How do providers perceive this service integration?

• Specifically, CRE/RT vs others?

c. How do patients and families perceive this service integration?

7. Thinking broadly, or specific to ARGI (but please indicate which you are talking about): what is the role of patient on your team?

1. Should that role be different?
2. Could it be better? How would that occur?

**iCOPE ARGI Patient Focus Group Guide**

SCRIPT:

[DO LOI AND CONSENT FIRST]

We would like to thank you for being here with us today and talking about your health care team.

We want to talk with you today to understand your experiences with your health care team.

This focus group is for you, the patients, to discuss together the care that you receive from your health care team and from any other care provider you have in this family health team. We are here to keep the discussion moving along. There are no wrong answers. During the focus group, we will take some time for you to draw out your team on a piece of paper and then after the focus group, we ask that you fill out a survey.

A reminder this focus group will be audio-recorded and there are some observers who will be taking notes, and will be around to help if you need.

Are there any questions?

We are going to start with talking about your health care team before we take some time to draw your health care team:

1. Describe your health care team.

a. Who do you have appointments with?

1. Draw your health care team.
2. Tell us about the care you receive from your HCT.
3. Your respiratory therapist.
4. Your Doctor
5. Other health care providers

4. How does the care you receive here compare to your hopes and expectations or any other care that you have received?

5. What could be done to better prepare you for managing your COPD?

a. What do you think is missing or would improve your care?

6. Please think back over the last 6 months and think about the care you have received from your RT for your COPD. Please rate the care that you have received over the past 6 months. Overall, did you find your care to be:

7. What is your role on the team?

1. How do you participate in your care?

8. Is there anything else that you would like to add to our discussion today?

PROBE: Looking back at the map that you drew earlier, is there anything that you would change about it?

**ARGI Provider Interview Guide**

Script

Hello

May I please speak with [Insert participant’s name]. *If they are not available, a message will not*

*be left.*

My name is [Research Assistant]. I am a research assistant working with Dr. Shannon Sibbald from Western University. I am assisting Dr. Sibbald today with conducting phone interviews with providers working with the respiratory therapists in your FHT.

Thank you for agreeing to participate in this interview. Is this time still convenient for you?

Before we begin, I need to ensure you have read the letter of consent, and have signed the consent form. I believe that [Name of ARGI Contact] has provided you with a copy of the study’s letter of information and consent. Do you have any questions about the information in the letter?

*[If already have consent]* Thank you for taking time to send in your sign consent form – we have received it.

*[If do not have consent]* We have not yet received your consent, please email or fax your consent as soon as possible so that we can use the information from this interview in our research.

Today we will have a short interview to better understand the respiratory team that works in your FHT, and how you, a provider, has been impacted by this team. The interview will be audio- recorded. Your participation in this study is voluntary, and you can decide to stop at any time. Everything that you say will be confidential and all data collected will be anonymous.

If you have any concerns with this interview or this study, the contact information for the principle investigator, Dr. Shannon Sibbald, or the ethics board at Western University are listed on the last page of the letter of information.

Do you agree to be audio-recorded? [begin audio-recording]

Do you agree to consent to this interview?

1. If you could explain a bit about yourself and the work that you do in the Thameview FHT.

2. Tell me about your experiences working with the RTs in your clinic.

a) Probe: Are you aware of the larger RT structure known as ARGI? Please tell me about your knowledge and experience with ARGI.

3. How does working with the RTs impact the way you practice?

a) PROBE: How does working with ARGI impact your practice?

4. How might you improve the ARGI RT model to better meet the needs of your practice?

5.  If ARGI were to be adapted to another FHT, what advice would you give?

a) PROBE: to the RTs? Docs? Other allied health professionals? EDs? so that this service could be used to its fullest?

References

Consolidated Framework for Implementation Research Booklet, (2018).

Retrieved from http://cfirwiki.net/guide/app/index.html#/

Davy, C., Bleasel, J., Liu, H., Tchan, M., Ponniah, S., & Brown, A. (2015). Factors influencing the implementation of chronic care models: A systematic literature review. *BMC Family Practice*, *16*(1). doi:10.1186/s12875-015-0319-5

Peer Implementation Literature Review/Summary Sheet

Tremblay, D., Touati, N., Roberge, D., Breton, M., Roch, G., Denis, J. L. & Francoeur, D. (2016). Understanding cancer networks better to implement them more effectively: a mixed methods multi-case study. *Implementation Science*, *11*(1), 1.


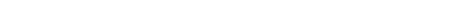


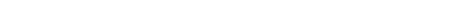

Supplement: Supplementary file 1 — Additional file 1. Patient & Provider Focus Group/Interview Guides. [file 12913_2020_5565_MOESM1_ESM.docx]
